# Supplementary material for: Modelling discrete states and long-term dynamics in functional brain networks
Source: Imaging Neurosci (Camb). 2026 May 26;4:IMAG.a.1237. doi: 10.1162/IMAG.a.1237 (PMC13214575; doi:10.1162/IMAG.a.1237)
Supplement: Supplementary Material [file IMAG.a.1237_supp.pdf]

Supplementary Materials

S.1 Model Training Details and Key Hyperparameters

S.1.1 Additional Techniques for Model Training

To facilitate faster and more stable convergence of DyNeStE, we adopted several additional training strategies. These techniques were designed to mitigate common issues found when training a VAE with RNNs and discrete latent variables.

Adaptive Optimisation Strategies

Three adaptive mechanisms are incorporated into the training process:

- **KL annealing:** When training a VAE with RNNs, the KL divergence term can dominate the loss and force the model to drive the variational posterior distribution close to the prior, resulting in a negligible KL loss (Bowman et al., 2015). To address this problem, we apply KL annealing to the objective function:

$$\mathcal{L} = -LL + \beta \cdot KL \tag{1}$$

  where β is a KL annealing factor that starts at 0 and gradually increases to 1 during training. This allows the encoder to first learn informative latent representations before aligning the variational distribution with the prior.

  The KL annealing schedule follows a hyperbolic tangent curve such that

$$\beta = 0.5 \cdot \tanh\left(\frac{a_s(n_{\text{epoch}} - 0.5 \cdot n_{\text{anneal}})}{n_{\text{anneal}}}\right) + 0.5 \tag{2}$$

  where a_s is the annealing sharpness, n_{epoch} is the total number of epochs, and n_{anneal} is the number of annealing epochs.

- **Gumbel-Softmax temperature annealing:** While Maddison et al. (2017) fixed the Gumbel-Softmax temperature and reported minimal benefits for their experiments, Jang et al. (2017) employed a temperature schedule when training VAEs with the Gumbel-Softmax distribution. In our experiments, temperature annealing was essential for achieving stable and better model performance. We used an exponential decay schedule:

$$\tau(\text{epoch}) = \max(\tau_{\text{end}}, \tau_0 \cdot \exp[-r_\tau \cdot \text{epoch}]) \tag{3}$$

where τ_0 and τ_{end} are the initial and final temperatures, respectively, and r_τ is the decay rate. This schedule allowed the model to learn a continuous posterior at the beginning and gradually transition into a more categorical distribution.

- **Learning rate scheduling:** We applied an exponential learning rate decay to accelerate training in the beginning and enable finer adjustments towards the end:

$$\eta = \eta_0 \cdot \exp[-r_\eta \cdot \text{epoch}] \tag{4}$$

where η_0 is the initial learning rate and r_η is the decay rate. In practice, we only begin decaying the learning rate after KL annealing is complete (i.e., $\beta = 1$). Note that this schedule was applied for HMM training as well.

Gradient Clipping

Although the aforesaid adaptive strategies improve convergence speed and stability, simultaneously varying multiple parameters (β, τ, and η) can make the optimisation landscape more complex. For a categorical model with discrete latent variables like DyNeStE, the loss surface tends to become increasingly noisy and non-smooth over time, especially as the Gumbel-Softmax temperature is annealed.

In certain model runs, we observed sporadic gradient explosions that destabilised training. To mitigate this, we constrained the gradient norm to a fixed threshold, preventing excessively large updates. While gradient clipping was unnecessary in our simulated experiments, it was crucial when training on real datasets.

Multi-start Initialisation

Since DyNeStE is trained using stochastic gradient descent, all trainable parameters are initialised randomly. The initialisation scheme for these parameters is as follows:

- **Weights and biases of the model and inference RNN:** Initialised using Glorot initialisation (Glorot & Bengio, 2010).
- **Layer normalisation parameters:** Layer normalisation (Ba et al., 2016) is applied between each RNN layer and subsequent dense layer in both the model and inference RNNs. Its learnable shift and scale parameters are initialised to zeros and ones, respectively.
- **Weights and biases of the dense layers:** Initialised using Glorot initialisation.

- **State means and covariances:** Mean vectors are fixed to zero, and covariance matrices are initialised using flattened Cholesky factors of identity matrices, with a Gaussian error added to the diagonal.

Because the objective function of DyNeStE is non-convex, its optimisation process is sensitive to parameter initialisation. We have observed that different random initialisations can result in high run-to-run variability, with a model converging to different local minima across runs. To mitigate this issue, we employed a multi-start initialisation strategy.

In this approach[1], the model parameters are re-initialised multiple times. For each initialisation, the model is trained for a small number of epochs, after which the loss is evaluated. The initialisation that yields the lowest loss is selected for the main training. During this initialisation stage, neither the Gumbel-Softmax temperature nor the KL annealing factor is annealed. Empirically, we find that this choice yields improved model performance.

Observation Model Regularisation

When training DyNeStE on real MEG data, we apply an additional regularisation to the state covariance matrices of the observation model. Specifically, we impose an inverse Wishart prior on each state covariance D_k, adding its negative log-density to the total loss:

$$-\log p(D_k) = \frac{\nu + M + 1}{2} \log |D_k| + \frac{1}{2}\mathrm{tr}(\Psi D_k^{-1}) \tag{5}$$

where M is the number of channels, $\nu > M - 1$ is the degrees of freedom, $|\cdot|$ denotes the matrix determinant, and Ψ is a symmetric positive-definite scale matrix. The first term penalises large determinants, preventing covariances from inflating arbitrarily; the second term penalises small or ill-conditioned covariances, preventing them from shrinking toward singularity that could artificially increase the likelihood. This prior encourages each D_k to remain close to a target scale and shape specified by Ψ and ν, avoiding both excessive stretching and degeneration in extreme directions. The same covariance regularisation is applied for the HMM.

With the exception of the Gumbel-Softmax temperature annealing, all of these techniques have been applied in prior models; see DyNeMo (Gohil et al., 2022) and M-DyNeMo (Huang et al., 2025) for additional discussions on methodological details.

[1] For the HMM, we adopt a slightly modified initialisation procedure. State time courses are randomly sampled, and the state-specific mean vectors and covariance matrices are computed from these samples. These empirically estimated means and covariances are then used to initialise the corresponding model parameters for each state. All remaining parameters are initialised following the standard scheme described above.

S.1.2 Model Training Curves

The loss curves of DyNeStE and the HMM, trained on the Nottingham MEGUK dataset, are shown below. Each curve corresponds to the best-performing run selected from ten model runs.

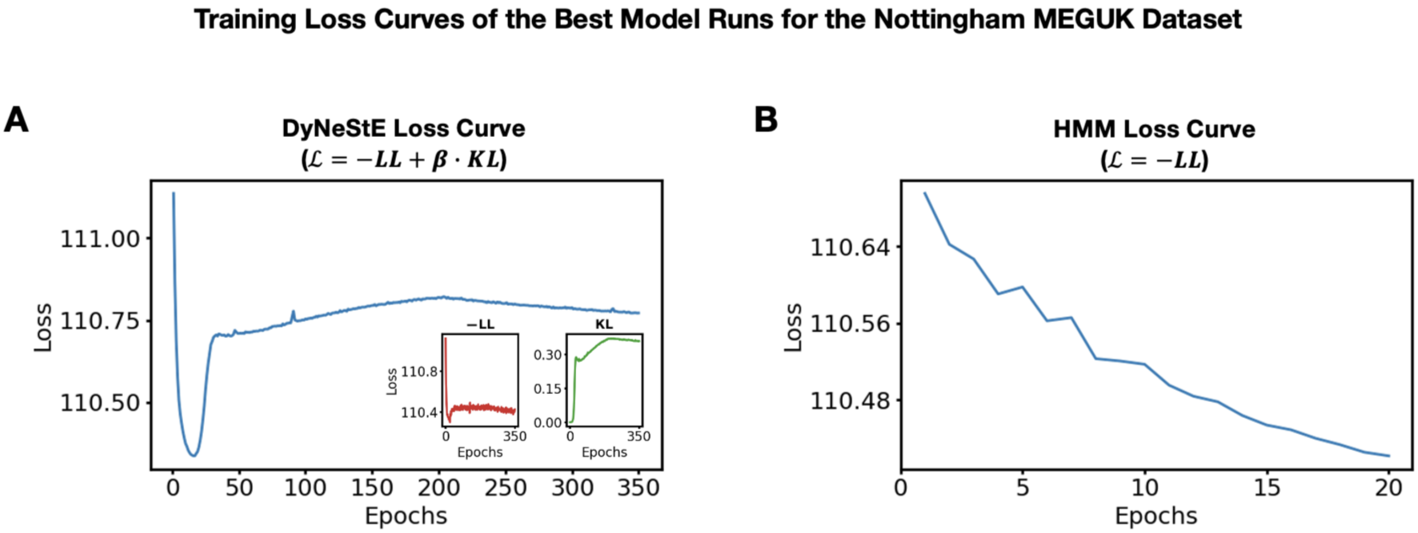

Figure S1: Training loss curves of DyNeStE and HMM. (A) Loss curve of DyNeStE (blue), where the loss is the variational free energy. The inset shows the negative log-likelihood (red) and KL divergence (green) terms separately. **(B)** Loss curve of the HMM (blue), where the loss is the negative log-likelihood.

To assess the stability of each model and characterise its variability across training runs, we extracted the final training losses from all ten runs and visualised them as a box plot. Note that the loss values of DyNeStE and the HMM are not directly comparable, as they are optimised using different objective functions.

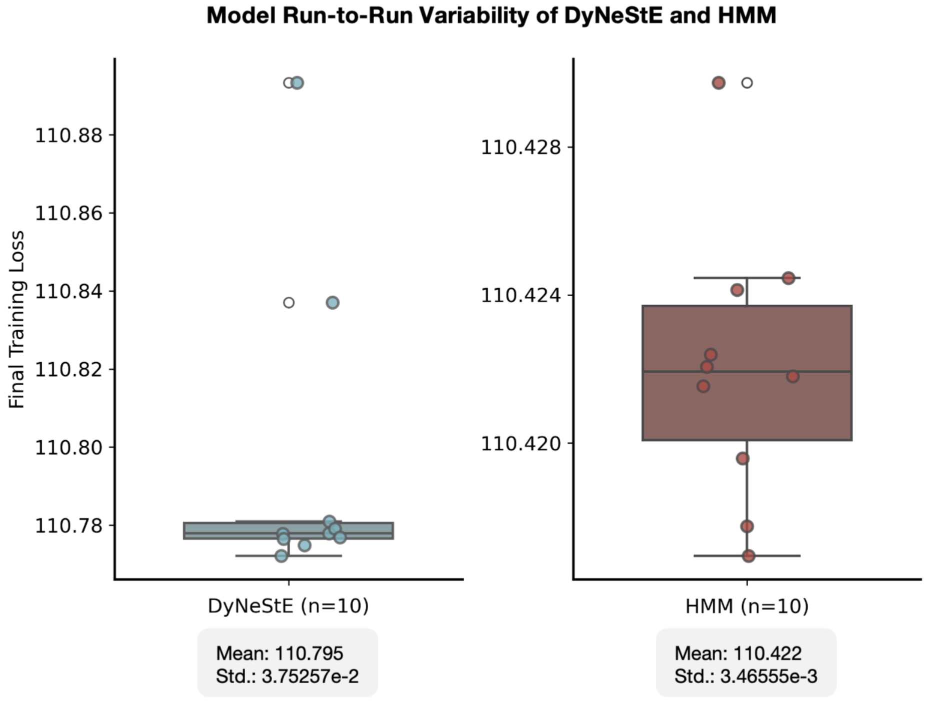

Figure S2: Run-to-run variability of final training loss for DyNeStE and HMM. Distributions of final training loss across 10 independent runs are shown as box plots for DyNeStE (left) and HMM (right). Coloured circles indicate loss values from individual runs, while open circles denote outliers. The mean and standard deviation of the final training losses for each model are reported under the corresponding box plot.

S.1.3 Model Hyperparameters

The hyperparameters of DyNeStE and the HMM used to train the models on the simulated and real MEG datasets are summarised in the tables below:

Table S1: (DyNeStE) Hyperparameters for Simulation and Real MEG Data

Hyperparameter	Simulation	Nottingham MEGUK
Number of states, K	3	12
Sequence length	200	200
Inference RNN† hidden units	128	128
Model RNN† hidden units	128	128
KL[1] annealing sharpness, a_s	10	10
KL annealing epochs, n_{anneal}	50	50
GS[2] initial temperature, τ_0	1	1
GS final temperature, τ_{end}	0.06	0.06
GS decay rate, r_τ	0.04	0.014
GS annealing epochs	120	350
Gradient clip (norm)	-	10
Batch size	16	128
Learning rate, η	5e-3	1e-3
Learning rate decay, r_η	0.01	5e-3
Number of epochs, n_{epoch}	120	350
Number of initialisations	5	10
Initialisation epochs	2	2

† Both the inference and model RNNs are a single-layer LSTM network.
[1] KL denotes the Kullback-Leibler divergence.
[2] GS denotes the Gumbel-Softmax distribution.

Table S2: (TDE-HMM) Hyperparameters for Simulation and Real MEG Data

Hyperparameter	Simulation	Nottingham MEGUK
Number of states, K	3	12
Sequence length	200	200
Batch size	16	256
Learning rate, η	0.01	0.01
Learning rate decay, r_η	0.1	0.1
Number of epochs, n_{epoch}	20	20
Number of initialisations	5	10
Initialisation epochs	2	2

Note that we set $K = 12$ for the real MEG datasets to allow direct comparison with previous studies that employed 12-state HMMs as their model specification (Higgins et al., 2021; van Es et al., 2025). For

the split-half reproducibility analysis in Fig. S5, the number of states was systematically varied across $K \in [4, 6, 8, 10]$.

In addition, the sequence length is chosen such that DyNeStE and HMM models have access to the same temporal context, ensuring a fair comparison. It has been tuned to optimise model performance under available GPU resources and compute time. For DyNeStE, the sequence length must be set to balance training stability and memory constraints against the need for a sufficiently long temporal window to capture long-range dependencies.

S.2 Dataset Details

The subject demographic information for the Nottingham MEGUK dataset is summarised in the figure below:

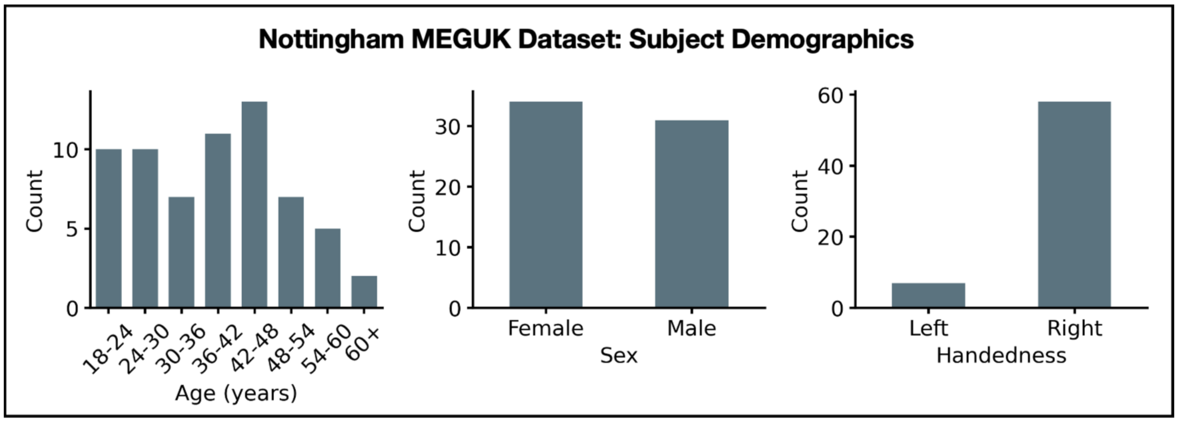

Figure S3: Nottingham MEGUK dataset subject demographics. Bar graphs depicting the histogram of participant ages (left), sexes (middle), and handedness (right).

S.3 Additional Figures for the Nottingham MEGUK Dataset

Here, we append additional results on the Nottingham MEGUK dataset.

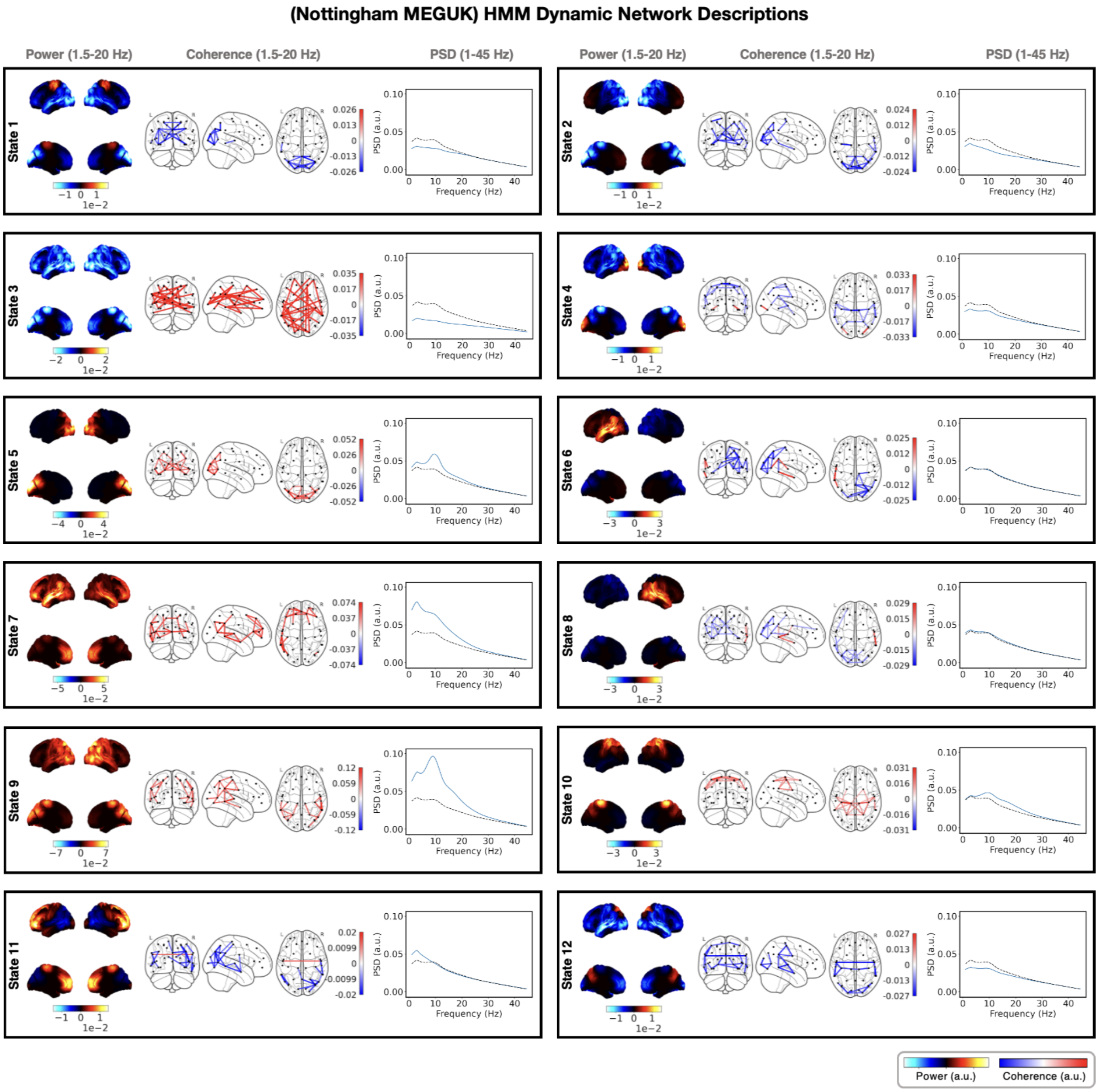

Figure S4: Dynamic resting-state MEG networks inferred by HMM. Using the HMM, twelve RSN states were identified from MEG recordings of 65 subjects. Each panel presents network descriptions of one state: the group-level power map (left), FC network (middle), and parcel-averaged PSD (right). The power maps display lateral and medial cortical surfaces at the top and bottom, respectively. The FC networks illustrate edges with the top 3% coherence values (regardless of sign). Both power maps and FC networks are shown relative to their average across all states. The PSD of each state (blue) is plotted alongside the state-averaged PSD (black dotted line).

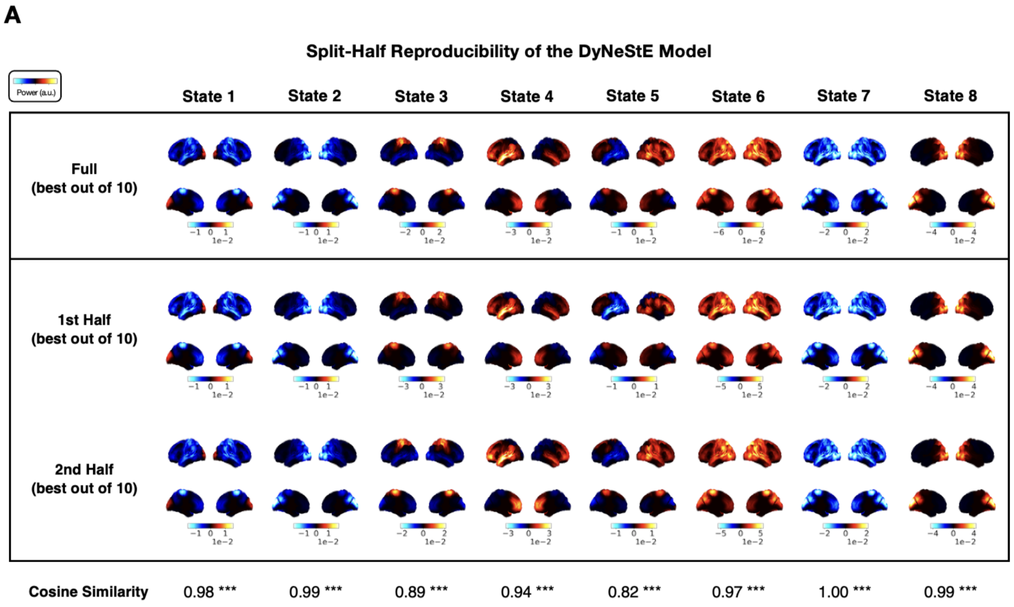

B

Cosine Similarities per Number of States

	State 1	2	3	4	5	6	7	8	9	10	11	12
12 States	0.747	0.866	0.997	0.887	0.867	0.742	-0.236	0.492	0.869	0.622	0.641	0.681
10 States	0.881	0.934	0.998	0.628	0.842	0.371	0.952	-0.673	0.635	0.995		
8 States	0.982	0.991	0.885	0.945	0.816	0.967	0.999	0.991				
6 States	0.851	0.938	0.944	0.955	0.959	0.998						
4 States	0.990	0.990	0.968	0.998								

FWER-Corrected Statistical Significance

***	**	*	n.s.
p < 0.05	p < 0.01	p < 0.001	p ≥ 0.05

Figure S5: Split-half reproducibility of DyNeStE across different number of states. DyNeStE was trained on two split-halves of the Nottingham MEGUK dataset with varying numbers of states as its hyperparameter, selecting the best model (out of 10 runs) for each subset. **(A)** Group-level power maps of 8 states inferred from the full dataset (top), first split-half (middle), and second split-half (bottom), each displayed relative to the mean power across all states. Cosine similarities between split-half power maps are reported for each state. **(B)** Cosine similarities between split-half power maps are shown for different number of states. Because the model was fitted with varying state numbers, the resulting states correspond to different brain networks, and their ordering does not necessarily align across model fits. Statistical significance was assessed using a max-statistic permutation test over the states (***: $p < 0.001$, **: $p < 0.01$, *: $p < 0.05$, n.s.: non-significant). Note that the test controls for family wise-error rate (FWER) by constructing the null distribution of max statistic over all states.

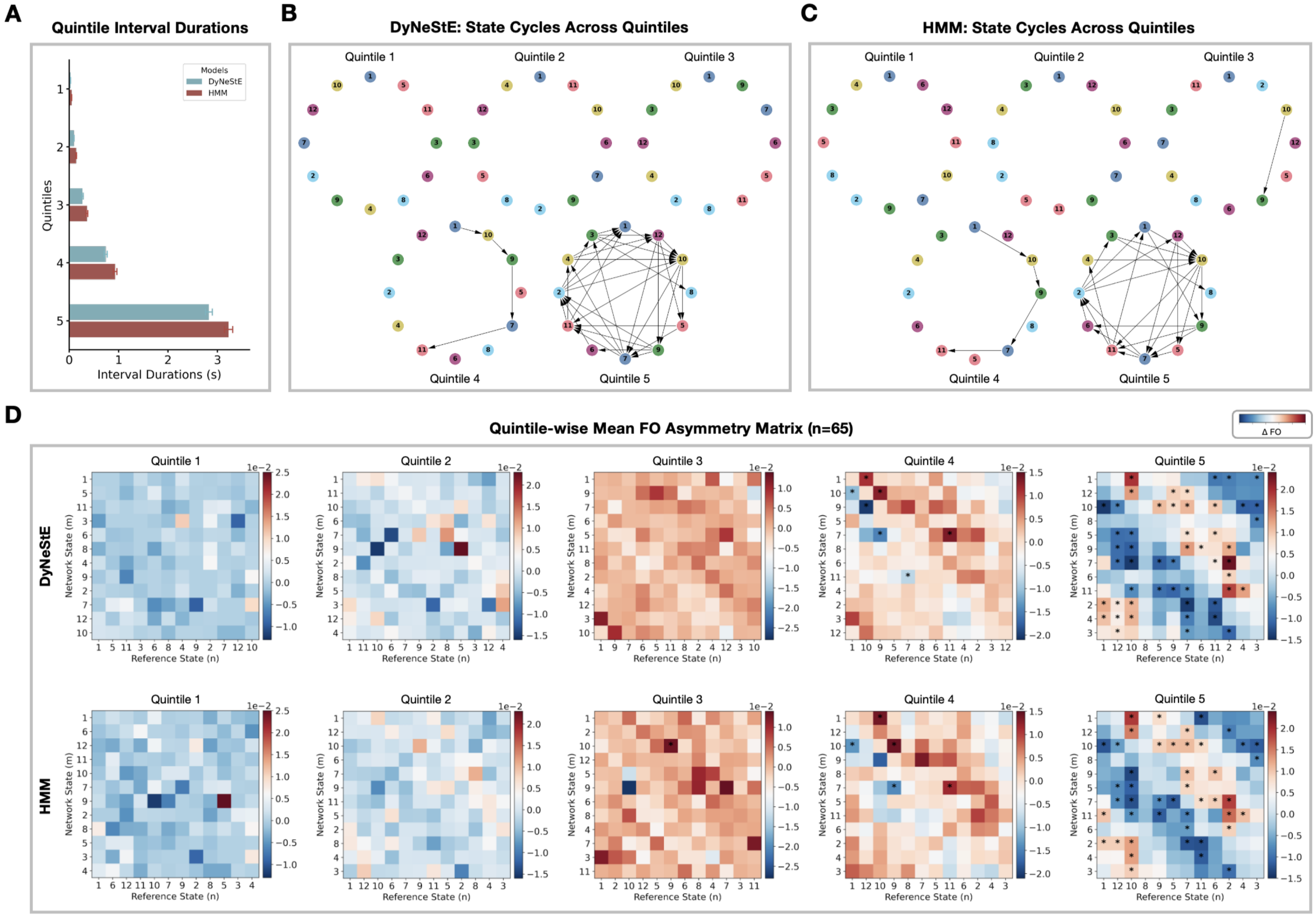

Figure S6: Quintile-wise cyclical organisation of state activations in observed data. (A) Distributions of inter-state interval durations, extracted from the inferred state time courses, are shown across quintiles. Mean values and standard errors are depicted as bar plots and error bars, respectively, for DyNeStE (blue) and the HMM (red). **(B)** Quintile-specific state cycles derived from the DyNeStE-inferred state time courses. **(C)** Quintile-specific state cycles derived from the HMM-inferred state time courses. **(D)** Group-averaged FO asymmetry matrices for each quintile, shown for DyNeStE (top) and the HMM (bottom). Asterisks mark statistically significant edges identified using a paired-samples t-test ($p < 0.05$, Bonferroni-corrected with $n = 132$ state pairs).

S.4 Additional Figures for the Replay Dataset

Lastly, we append additional results on the Replay dataset.

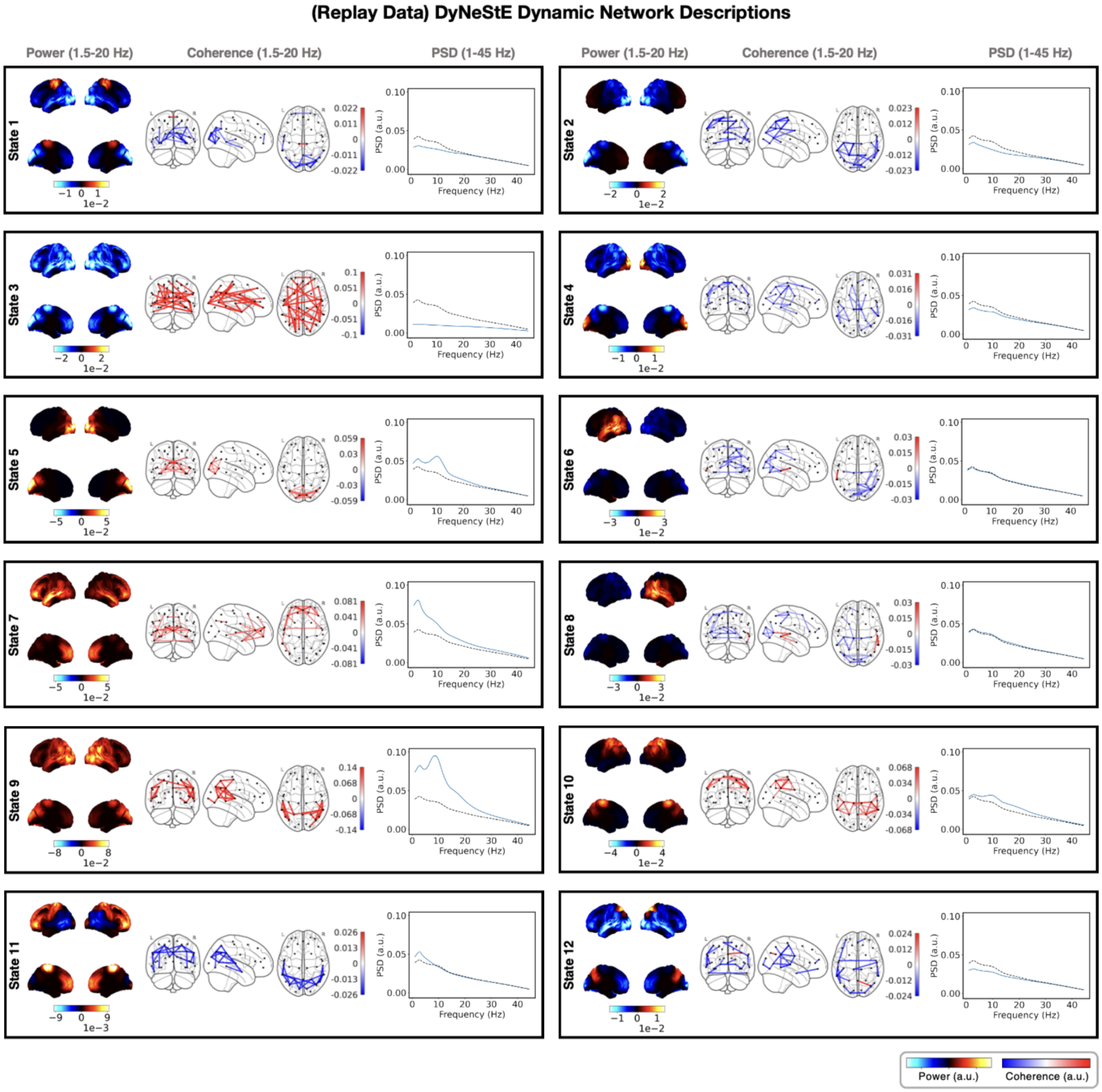

Figure S7: Dynamic resting-state MEG networks inferred by DyNeStE. Twelve RSN states were identified from MEG recordings comprising 42 sessions (21 subjects, two sessions each) in the Replay dataset using DyNeStE. Each panel presents network descriptions of one state: the group-level power map (left), FC network (middle), and parcel-averaged PSD (right). The power maps display lateral and medial cortical surfaces at the top and bottom, respectively. The FC networks illustrate edges with the top 3% coherence values (regardless of sign). Both power maps and FC networks are shown relative to their average across all states. The PSD of each state (blue) is plotted alongside the state-averaged PSD (black dotted line).

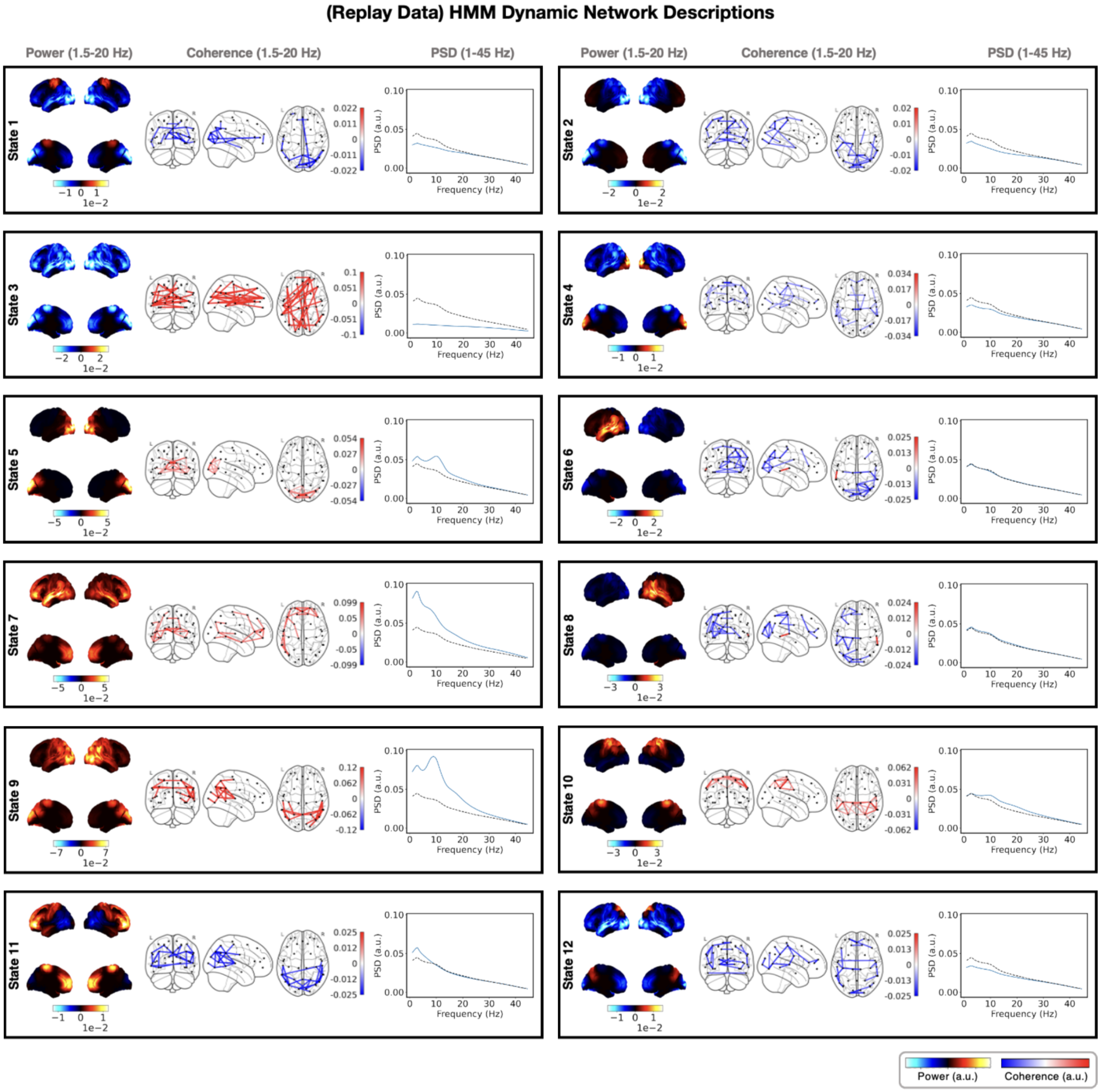

Figure S8: Dynamic resting-state MEG networks inferred by HMM. Twelve RSN states were identified from MEG recordings comprising 42 sessions (21 subjects, two sessions each) in the Replay dataset using the HMM. Visualisations are presented in the same format as in Figure S7.
